# Behavioral Strategies to Minimize Procedural Distress During In-Office Pediatric Tympanostomy Tube Placement Without Sedation or Restraint

**DOI:** 10.1007/s10880-021-09813-0

**Published:** 2021-08-31

**Authors:** Lindsey L. Cohen, Abigail S. Robbertz, Laura J. England

**Affiliations:** 1 Department of Psychology, Georgia State University, Atlanta, GA 30302-5010 USA; 2 Smith+Nephew, 155 Jefferson Drive, Menlo Park, CA 94025 USA

**Keywords:** Distraction, Myringotomy, Pediatric, Preparation techniques, Procedural distress, Tympanostomy tube placement

## Abstract

The purpose of this study was to evaluate behavioral strategies to minimize procedural distress associated with in-office tympanostomy tube placement for children without general anesthesia, sedation, or papoose-board restraints. 120 6-month- to 4-year-olds and 102 5- to 12-year-olds were treated at 16 otolaryngology practices. Mean age of children was 4.7 years old (SD = 3.18 years), with more boys (58.1%) than girls (41.9%). The cohort included 14% Hispanic or Latinx, 84.2% White, 12.6% Black, 1.8% Asian and 4.1% ‘Other’ race and ethnicity classifications. The in-office tube placement procedure included local anesthesia via lidocaine/epinephrine iontophoresis and tube placement using an integrated and automated myringotomy and tube delivery system. Behavioral strategies were used to minimize procedural distress. Anxiolytics, sedation, or papoose board were not used. Pain was measured via the faces pain scale-revised (FPS-R) self-reported by the children ages 5 through 12 years. Independent coders supervised by a psychologist completed the face, legs, activity, cry, consolability (FLACC) behavior observational rating scale to quantify children’s distress. Mean FPS-R score for tube placement was 3.30, in the “mild’ pain range, and decreased to 1.69 at 5-min post-procedure. Mean tube placement FLACC score was 4.0 (out of a maximum score of 10) for children ages 6 months to 4 years and was 0.4 for children age 5–12 years. Mean FLACC score 3-min post-tube placement was 1.3 for children ages 6 months to 4 years and was 0.2 for children age 5–12 years. FLACC scores were inversely correlated with age, with older children displaying lower distress. The iontophoresis, tube delivery system and behavioral program were associated with generally low behavioral distress. These data suggest that pediatric tympanostomy and tube placement can be achieved in the outpatient setting without anxiolytics, sedatives, or mechanical restraints.

Myringotomy and tympanostomy tube placement for recurrent acute otitis media or chronic otitis media with effusion is the most common ambulatory pediatric surgical procedure in the USA, accounting for 24% of all pediatric (0–15 years of age) ambulatory surgeries (Hall et al., 2017). The procedure involves making an incision in the tympanic membrane (TM) and inserting a tube to maintain ventilation and prevent recurrence of fluid. This surgery is typically brief and uncomplicated; given the extreme sensitivity of the eardrum and the mobility of young children, general anesthesia is employed. Unfortunately, most children as well as their parents have high distress associated with anesthesia (Chorney & Kain, 2009; Davidson & McKenzie, 2011; Fortier et al., 2010; Kain et al., 1999). In addition, following elective outpatient surgery, data indicate that children can experience short-term behavioral problems, such as post-surgical emergence delirium, sleep problems, and eating disturbances (Kain et al., 2006; Mason, 2017). Beyond emotional and behavioral issues, there can be complications with general anesthesia in pediatric patients, even with the brief anesthetic exposure required for a tympanostomy procedure, especially in young children (Ing et al., 2014; Wang et al., 2014; Zhang et al., 2015).. Although adverse effects of general anesthesia during tube procedures are uncommon, they can be severe, such as dysrhythmia (1.8%), severe airway obstruction (1.4%), laryngospasm (0.9%), blood oxygen desaturation (0.4%), and post-operative vomiting requiring treatment (0.4%) (Hoffman et al., 2002; Markowitz-Spence et al., 1990). In addition, a significant proportion of children undergoing general anesthesia may experience emergence delirium; Cravero et al. (2000) reported that 57% of children exposed to sevoflurane for tube placement showed emergence delirium, defined as 3 or more minutes of thrashing requiring restraint.

Concerns have also been raised regarding the potential long-term impact of general anesthetics on patients For anesthesia exposure in children under age 3, risks include language, cognitive, and behavioral disorders at age 10 (Ing et al., 2014). Increased risk has been noted in children up to age 4, with level of risk increasing with the amount of anesthesia exposure (Wang et al., 2014).

To address these concerns, a treatment package was developed that included a medical device, customized otic anesthetic, anesthetic delivery system, and a behavioral program. Smith+Nephew (Menlo Park, CA) developed a novel medical device and drug system (referred to as the “Tula® System”) to conduct pediatric myringotomy and tympanostomy tube placement in the outpatient physician office setting without requiring general anesthesia or sedation. The iontophoresis system used in conjunction with an otic anesthetic solution (TYMBION™, 2% lidocaine HCl and 1:100,000 epinephrine) was developed to provide numbing of the eardrum in approximately 10 min. The automated myringotomy and tube delivery system was created to allow physicians to rapidly place the tympanostomy tube. Given that prior data (Zeiders et al., 2015) indicated an average procedure time of 32 min for numbing and tube placement, that the patient should remain still and in the medical chair, and that the typical target population includes very young children, pediatric agitation or distress was expected.

A clinical psychologist (lead author) developed the behavioral program to optimize cooperation and minimize procedural distress. Distress—an umbrella construct composed of fear, agitation, anxiety, and pain—was targeted consistent with a common conceptualization in the pediatric procedure literature (e.g., Cohen et al., 2004, 2017; Siegel, 1988). The program was grounded in the rich literature documenting successful cognitive-behavioral strategies for pediatric procedural distress management (e.g., Cohen et al., 2017). For example, data generally suggest that parent presence is optimal if they are trained in strategies to minimize their child’s distress, such as avoiding certain behavior (e.g., excessive reassurance, criticism) and engaging in coping promoting behavior (e.g., distraction; Cohen et al., 2020). Further, studies have shown that preparation is effective and should include information about procedural steps as well as sensory experiences (Jaaniste et al., 2007). When conducting the procedure, evidence-based strategies include providing information in non-emotive tones; using frequent and varied distraction prior to, during, and immediately following stressful procedural junctures; reinforcing cooperative and calm behavior; and highlighting and reward positive child behavior following the event to encourage positive memories (e.g., Cohen et al., 2020).

The program included specially developed stimuli. For example, the psychologist consulted on the development of an illustrated child storybook developed titled, “We’re going to the ear doctor!” The story detailed the procedural steps using reframing. For example, the child in the story “feels the squish of cold medicine in her ears as the doctor fills them. It feels like a bubble bath in her ears!” Parents were provided additional coaching tips in a training booklet and stickers and a sticker chart to reinforce behavior throughout the procedure. The psychologist trained all physicians in advance and provided them a booklet detailing how to provide information, distraction, and reinforcement of appropriate behavior to the children. The booklet had evidence-based information (e.g., suggested child-appropriate language, trouble-shooting tips). In addition, the psychologist adopted a train-the-trainer approach, and taught the physicians how to guide, support, and prompt parents to engage in appropriate child coaching throughout the procedure.

A preliminary study using earlier generations of the devices, drug solution, and behavioral program enrolled nine physicians and seventy children (mean age 7.0 years; Zeiders et al., 2015). The pilot behavioral program was described in Cohen et al. (2015) and shared the principals and strategies with the current program; updates to the program included revised and improved preparation materials, updated distraction stimuli, and other modifications for younger patients. In the preliminary study, there were no serious adverse events and tube placement was successful in 96.6% (114/118) of ears (Zeiders et al., 2015). Observational scoring suggested minimal distress throughout the procedure (Cohen et al., 2015). The preliminary study was limited in that there were few young patients (15 children were 3 years or younger, including only 2 children under the age of 1) and a small cohort of 9 physicians. Thus, in addition to evaluating improved systems and an updated behavioral program, the current study enrolled a population more typical of the patient population receiving tubes and included larger cohort of physicians.

The current study is (a) a companion to the study by Lustig et al. (2020), which focused on tube placement success (i.e., tubes were placed in 87% of subjects) and safety outcomes (i.e., no serious adverse events), and (b) an extension of the earlier study of the behavioral program (Cohen et al., 2015). Our initial analyses focused on differences in distress by gender and age. As data suggest little difference in pain report or display by gender prior to puberty (e.g., Boerner et al., 2014), we did not anticipate finding gender differences. We did expect younger children to report and display higher distress than older children, which would be consistent with the literature (e.g., Cohen et al., 2017). Our primary hypothesis was that patients would report and display minimal distress associated with the tube placement procedure.

## Method

### Study Design and Oversight

The trial (NCT03323736, OTTER) adhered to a protocol approved by the FDA and institutional review boards, with additional medical oversight by an independent panel including an audiologist, general otolaryngologists, a pediatric otolaryngologist, and a pediatric emergency medicine physician specializing in child pain. The independent panel was not involved in any aspect of study development or evaluation; they were solely in place if there were extreme medical or tolerability issues.

The study used a prospective, multicenter, single-arm design to examine children’s behavioral distress during the in-office tympanostomy tube procedures. A control group was not included as it would be unethical to conduct tube placement on awake children without local anesthesia or behavioral program.

### Participants

Results from prior studies described above (Cohen et al., 2015; Zeiders et al., 2015) suggested that younger children may be more challenging in-office patients than older children due primarily to behavior. In addition, most pediatric tube procedures occur in younger children (e.g., 0–4 years of age). The study was therefore designed to ensure sufficient enrollment across all ages and to evaluate results from the 6-month to 4-year-olds and the 5- to 12-year-olds separately.

Pediatric participants consisted of 222 patients diagnosed with chronic otitis media with effusion and/or with recurrent acute otitis media and indicated for tube placement surgery per the American Academy of Otolaryngology—Head Neck Surgery Clinical Practice Guidelines (Rosenfeld et al., 2013). The participating physicians identified 6-month- to 12-year-old patients who required tympanostomy surgery and who demonstrated adequate minimal cooperative behavior (i.e., child could remain still during a brief routine ear exam and cleaning) for an office tube placement procedure. Parents consented and children assented (as appropriate) prior to enrollment. Exclusion criteria included conditions that could interfere with safe or effective placement of tympanostomy tubes, such as atelectatic TM, TM perforation, damaged ear canal skin, or allergy to the anesthetic. Most study patients (91.4%) were indicated to receive bilateral tubes. Children were on average 4.7 years old (SD = 3.18 years), with 120 6-month- to 4-year-olds (*M* = 2.3, SD = 1.38) and 102 5- to 12-year-olds (*M* = 7.6, SD = 2.10). Additionally, these age groups were determined based on the reliability of the recommended measures of pain for specific ages (Cohen et al., 2008; Tsze et al., 2018; von Baeyer & Spagrud, 2007). For children in the 0- to 5-year-old group, 19.2% patients had a prior operating room (OR) tube placement compared to 43.1% children in the 5- to 12-year-old group. There were slightly more boys (*n* = 129, 58.1%) than girls (*n* = 93, 41.9%). Regarding ethnicity and race, 84.2% were White, 14% were Hispanic or Latinx, 12.6% were Black, 4.1% were ‘Other,’ and 1.8% were Asian. A minimum of one caregiver was present at time of the procedure, typically the child’s mother.

Twenty physicians at sixteen otolaryngology practices in the US and Canada recruited patients and conducted the office tube placement procedures over a 15-month period. As part of training, each physician performed at least two procedures with the automated tympanostomy device in the operating room with pediatric patients under general anesthesia and another two procedures in the office setting with the full Tula system including the iontophoresis system and the tympanostomy device prior to enrolling pediatric patients for the study. A subset of physicians had prior investigational study experience with the office tube placement procedure for adults (9 physicians) or children (3 physicians). The psychologist and their staff reviewed videos of the procedure to ensure adherence to study protocol.

### Measures

#### Self-Reported Distress

The faces pain scale-revised (FPS-R) is a single-item pain intensity scale that allows children to select 1–6 faces with increasing expressions of pain that best represents their experience (Hicks et al., 2001). The scale is scored from 0 (no pain) to 10 (very much pain). Children 5–12 years old rated their pain with the FPS-R prior to initiation of the procedure (baseline), immediately after tube placement was complete when the most acute pain was anticipated, and at 5 min following the procedure to assess the temporal extent of the discomfort, if experienced. The FPS-R is commonly used and is recommended by the PedIMMPACT consensus group for assessment of acute pain intensity associated with procedure-related, post-operative and disease-related pain (McGrath et al., 2008); however, there is acknowledgment that ratings reflect affective (e.g., anxiety) and sensory aspects of the experience (i.e., distress; Champion, 1998). The scale has strong psychometric support for self-reported pain intensity (Hicks et al., 2001). The FPS-R is discouraged for children under the age of 5 because they tend to select the extreme faces, which underestimates or inflates pain ratings (Arts et al., 1994; Chambers et al., 2002). The FPS-R has been used across racial and ethnic groups (e.g., Naegeli et al., 2018; Tsze et al., 2013). Finley et al. (2009) conclude that no evidence suggests that the FPS-R should not be used universally across cultures. Summary FPS-R scores are provided as an indicator of tolerability in the companion paper (Lustig et al., 2020), but more detailed self-reported distress as well as relevant correlations are included in the current results.

#### Observed Distress

The FLACC (face, legs, activity, cry, consolability) provided a measure of observer-rated overt behavioral distress (Merkel et al., 1997). The FLACC is a 0–10 pediatric observational measure that allows observers to score the distress of patients as 0, 1, or 2 based on descriptors associated with the scoring system for facial expression, leg position, activity, crying, and responsiveness to being consoled. For example, in the Face dimension, a score of ‘0’ corresponds to ‘No particular expression or smile’, a score of ‘1’ reflects ‘Occasional grimace or frown, withdrawn, uninterested,’ and a score of ‘2’ corresponds to ‘Frequent to constant quivering chin, clenched jaw.’ The PedIMMPACT consensus group recommends the FLACC for studies examining pediatric procedural distress (McGrath et al., 2008). The scale is frequently used and has strong psychometric support (Crellin et al., 2018; Nilsson et al., 2008).

The entire study procedure was video-recorded, the disclosure of which was included in the consenting process, and procedural recordings were supplied to the psychologist’s laboratory for coding. FLACC was coded using the method described in Gomez et al. (2013) and Voepel-Lewis et al. (2002). Three research assistants, blind to study aims, conducted FLACC coding of video-recordings, under the supervision of the psychologist. Research assistants were trained to use the FLACC coding by practicing on prior tube placement study data to ensure interrater reliability. Once at least 80% of the scores were identical for the research assistants’ independent coding of videos, they initiated scoring of study video data. Throughout coding of study data, research assistants were assigned batches of 10 subjects with 20% overlap (2 of the 10 subjects assigned to 2 different coders). The 2 research assistant coders were not aware of which videos were unique or redundant. The redundant scores were checked for score agreement and discrepancies, if any, were resolved via discussions as well as coding by the psychologist as a standard. In addition, reliability analyses utilizing the calculation of a weighted Cohen’s Kappa statistic were conducted to determine agreement across all FLACC categories (i.e., faces, legs, activity, cry, consolability). Once coders were in agreement and Kappa was 0.6 or greater, the next batch of subjects was assigned to the coders.

FLACC scores were assigned for each of 5 phases to assess the unique experiences for each procedure step, and for relative context for the investigational iontophoresis and tube placement procedures. These phases were determined a priori based on a discussion among the psychologist, device development team, and expert otolaryngologists. Phase 1 consisted of pre-procedure otoscopy, a routine ear examination with FLACC evaluated from 30 s prior to the speculum entering the ear canal until 60 s after the ear exam. Phase 2 included earset installation and filling comprised of insertion of a soft earplug and filling the ear canal with otic anesthetic solution which can yield a surprising and ticklish sensation with FLACC evaluated from 30 s prior to the iontophoresis earset first touching the child’s head until 60 s after otic anesthetic solution filling of the external ear canal was complete. Phase 3 consisted of iontophoresis during which electrical current is delivered to the ear in the presence of the anesthetic solution to numb the ear drum with FLACC evaluated from the initiation of the iontophoresis process until 60 s after the otic anesthetic solution was removed from the external ear canal. Phase 4 included tympanic membrane anesthesia assessment, in which the ear drum is lightly touched with a dull otologic instrument to determine if the TM is insensate or not, and tube placement during which a myringotomy incision is made and the tube placed across the ear drum. Phase 4 FLACC evaluation was initiated from the moment the speculum enters the ear until 60 s after the automated tympanostomy device is withdrawn from the external ear canal. Phase 5 consisted of the 3-min period post-procedure, from the end of the prior phase until 180 s later. In addition, a sixth FLACC score was assigned as an overall score for the entire procedure. A mean FLACC score for each procedural phase and overall was calculated from the average FLACC scores of the study participant population.

## Results

Most patients (91.4%, 203/222) were medically indicated to receive bilateral tubes, and tubes were successfully placed in the majority of the sample. Successful tube placement was achieved for 86% (175/203) of bilaterally indicated participants, and 100% (19/19) unilateral patients (Lustig et al., 2020). As per the FDA-agreed upon protocol, patients were excluded from FPS-R or FLACC analyses if they did not successfully complete the procedure for all indicated ears. Reasons for incomplete procedures included excessive movement or other behavior that interfered with completion (*n* = 11), inadequate anesthesia as determined by the tympanic membrane anesthesia assessment (*n* = 7), discomfort/anxiety (*n* = 4), anatomic challenges (*n* = 3), intolerance of iontophoresis (*n* = 2), and partial tube medialization (*n* = 1). The following results reflect the 194 children with successful tube placements. Per protocol and so that reports reflected the entire procedure, no FPS-R scores were collected from the 11 children for whom tube placement was incomplete. Similarly, FLACC analyses included only patients with successful procedures given that unsuccessful procedures would be dissimilar (e.g., truncated intervention, shortened procedure). Post hoc analyses revealed no differences in FLACC scores between these 194 successful cases and the full sample. FPS-R scores are reported from 5- to 12-year-old patients except two children that were incapable of self-reporting FPS-R scores; one child had autism, one had spinal muscular atrophy.

**Fig. 1:**
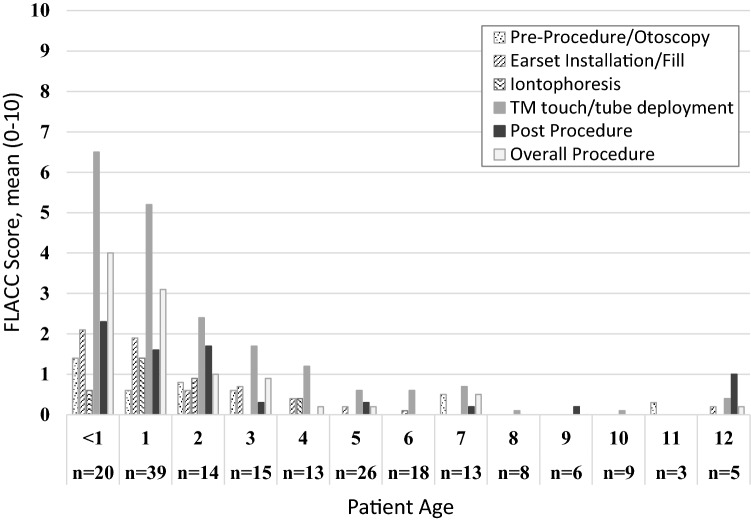
FPS-R scores by procedural phase and age

FPS-R scores ranged from 0–10 for all phases, and FPS-R baseline pain score mean was 0.59 (*n* = 88, SD = 1.46), post-tube placement mean score was 3.30 (*n* = 89, SD = 3.39), and 5-min post-procedure mean score was 1.69 (*n* = 89, SD = 2.43) (Table 1). There were no significant differences in males’ and females’ tube placement scores, *p* > 0.05. In addition, age was not correlated with tube placement FPS-R score, *p* > 0.05 (Fig. 1).

**Table 1:** FPS-R scores* by procedural phase and age

	Pre-procedure	Post-tube placement	5-min post-procedure
	*M* (SD)	*M* (SD)	*M* (SD)
All Participants	0.6 (1.5)	3.3 (3.4)	1.7 (2.4)
5 years	0.7 (2.1)	4.1 (3.9)	1.5 (2.3)
6 years	0.5 (1.1)	2.4 (2.8)	1.4 (1.8)
7 years	0.5 (0.9)	4.1 (2.8)	1.6 (2.4)
8 years	1.0 (1.9)	3.3 (3.7)	3.0 (3.9)
9 years	0.9 (1.6)	3.7 (5.0)	1.7 (2.7)
10 years	0.4 (1.3)	2.0 (2.2.)	1.1 (1.5)
11 years	0.0 (0.0)	1.3 (1.2)	2.0 (0.0)
12 years	0.7 (1.6)	3.2 (3.9)	2.8 (4.1)

*The FPS-R scale is scored 0–10 with higher scores indicating higher distress

**Fig. 2:**
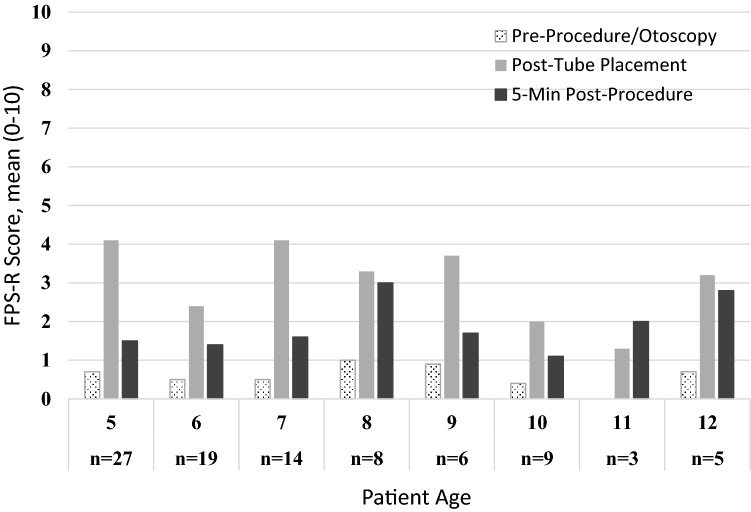
FLACC scores by procedural phase and age

FLACC was scored for all patients except 5 due to technical issues resulting in no video recording. FLACC scores ranged from 0–10 for all phases except the pre-procedure otoscopy, which ranged from 0–8. Across all 5 procedural phases and the overall impression, the average FLACC behavioral distress score ranged from 0.1 to 4.0 (Table 2). FLACC pre-procedure otoscopy mean score for all subjects was 0.4 (*n* = 179, SD = 1.5), earset install and filling mean score was 0.8 (*n* = 187, SD = 2.2), iontophoresis mean score was 0.5 (*n* = 188, *SD* = 1.6), TM tap and tube placement mean score was 2.4 (*n* = 188, SD = 3.3), post-procedure mean score was 0.8 (*n* = 188, SD = 1.7), and overall procedure mean score was 1.3 (*n* = 189, SD = 2.6). Breakdown of FLACC scores for each age group is further described in Table 2. Given the overall low phase scores, statistical analyses were only conducted for the tube placement phase which exhibited the highest FLACC scores. There were no significant differences in tube placement FLACC scores between boys (*n* = 110, *M* = 2.14, SD = 3.16) and girls (*n* = 78, *M* = 2.71, SD = 3.48), *p* > 0.05. There was a significant correlation between FLACC and age for the tube placement phase, *p* < 0.01, with younger participants having higher FLACC scores than older participants (Fig. 2).

**Table 2:** FLACC scores* by procedural phase and age

	Pre-procedure otoscopy	Earset installation and filling	Iontophoresis	Eardrum tap and tube placement	Post-procedure	Overall procedure
	*M* (SD)	*M* (SD)	*M* (SD)	*M* (SD)	*M* (SD)	*M* (SD)
All participants	0.4 (1.5)	0.8 (2.2)	0.5 (1.6)	2.3 (3.3)	0.8 (1.7)	1.3 (2.6)
6 months to 4 years	0.7 (2.0)	1.4 (2.8)	0.8 (2.1)	4.0 (3.6)	1.3 (2.1)	2.3 (3.2)
5 to 12 years	0.1 (0.7)	0.1 (0.5)	0.0 (0.1)	0.4 (1.2)	0.2 (0.9)	0.1 (0.8)

*The FLACC scale is scored 0–10 with higher scores indicating higher distress

Given the differences regarding the timing of data collection for FPS-R and FLACC scores, comparison of FPS-R and FLACC scores could only be assessed for the tube placement phase. At tube placement, there was not a significant correlation between FPS-R and FLACC scores, *p* > 0.05, for children ages 5–12.

## Discussion

Results were generally consistent with expectations. Although there were no gender differences, older children displayed lower behavioral distress. In terms of primary aims, distress was overall low as hypothesized, but there was variability in outcomes. For example, children under 12 months old had relatively higher distress, especially at the tube deployment phase. This was expected given that we were requiring that these infants stay in an unfamiliar environment (e.g., relatively cold room, bright lights), remain supine for extended periods of time, and healthcare professionals were touching and looking in the child’s sensitive ears. In addition, at tube deployment there is a loud clicking sound that can be startling and frightening; this auditory event alone could explain elevated distress behavior. Anecdotal reports from the coders corroborated the impressions that distress in young patients was related to these environmental factors rather than the myringotomy and tube deployment procedure.

As expected, older children had minimal to any reported pain and displayed little distress. Specifically, the self-reported pain score means fell in the “mild” pain range based on research aiming to contextualize 0–10 pain scores (e.g., Tsze et al., 2018). Acknowledging important differences based on setting, population, and procedure, the FPS-R pain scores collected in this study do not appear to be clinically meaningfully different than mean scores reported from similarly aged patients following surgery (2.93; von Baeyer et al., 2009), receiving immunizations (range 3.0–6.6; Berberich & Landman, 2009; Boivin et al., 2008), dental injections (range 3.0–6.3; Asvanund et al., 2015; Deepak et al., 2017; Kamath, 2013), IV cannulation (3.9; Taddio et al., 2005), venipuncture (range 3.3–6.5; Inal & Kelleci, 2012; Karakaya & Gozen, 2016), and ear piercing (3.9; Hicks et al., 2001). The mean FPS-R score reported five minutes after tube placement was 1.69, suggesting that distress was transient.

Overall, the mean FLACC scores in this young sample were generally in a range that was comparable to mean FLACC scores reported for 4- to 6-year-olds receiving immunization injections (Berberich & Landman, 2009; Franck et al., 2015), 4- to 13-year-olds receiving dental injections (Asvanund et al., 2015; Pala et al., 2016; Thoppe-Dhamadharan et al., 2015), 1- to 10-year-olds undergoing venipuncture (Gupta et al., 2014; Minute et al., 2012), and 2- to 7-year-olds undergoing allergy skin prick test (Goldberg et al., 2014). A review of the literature indicates that younger children generally display higher behavioral distress than older children, consistent with the findings in this study (von Baeyer & Spagrud, 2007; Young, 2005).

The in-office tympanostomy procedure FLACC can be compared to post-operative distress after OR-placed tympanostomy tubes. Mean FLACC scores in the recovery room after OR-placed tympanostomy tubes from two separate studies ranged from 2.0 to 4.8, varying with use of pre-operative and intra-operative medications such as midazolam, dexmedetomidine, acetaminophen, fentanyl, and morphine (Hippard et al., 2012; Dewhirst et al., 2014). In a large study of 3669 children, 21% of patients in the recovery after OR-placed tubes had FLACC scores of 7–10, despite pre-operative midazolam and intra-operative fentanyl or ketorolac (Stricker et al., 2017). It is not known if this post-operative distress is due to excessive pain from the tube placement or related to the after-effects of the anesthetic (i.e., emergence agitation). In contrast, this office tube placement study using local anesthesia had low mean FLACC scores 3-min post-procedure consistent with low post-operative distress.

The lack of correlation between FPS-R and FLACC scores is notable. Data within a single study and across studies suggest that associations between FPS-R and FLACC range from no significant relationship to strong correlations (da Silva et al., 2011; Emmott et al., 2017). Consistent with the perspective that self-report and observer-report scales might be reflecting different—and valuable—aspects of the same event (Cohen et al., 2008), our findings underscore that we assessed different outcomes in line with the long-standing recommendation that pediatric procedural pain assessment be conducted in a multimethod manner (American Academy of Pediatrics Committee on Psychosocial Aspects of Child and Family Health; Task Force on Pain in Infants, Children and Adolescents; 2001; Cohen et al., 2020; McGrath & Gillespie, 2001).

It is important to note that whereas the majority of children reported and displayed minimal distress, a subset of patients reported or exhibited high distress. This distress was transitory as shown by low scores within 3-min post-procedure; peaked during tube delivery; and while discomfort may have been associated with tube insertion, anecdotally the distress often occurred when a child was encouraged to lay in a supine position, when the head was stabilized, or when the click of the device surprised the child. The broader pediatric procedural literature indicates that children exhibit higher distress when laying on their back during medical procedures (Bice & Wyatt, 2017; Cohen, 2008; Sri Rahyanti et al., 2017).

Particularly in pediatric patients, variability in pain and distress is anticipated. This is believed to be due, in part, to the fact that children have challenges in distinguishing pain from emotional aspects of distress. FLACC scores have been shown to range from 0 to 10, associated with simple palpation of a vein in children 1- to 6-year-olds (Lunoe et al., 2015), and Koc and Gozen (2015) showed that 83% of FLACC scores for infants (1 to 12 months old) were greater than 4 after simple physical measurements (height, weight, head circumference, and oxygen saturation). Tsze et al. (2018) demonstrated that 32% of children (mean age of 8.6 years old) who were not in any pain reported an FPS-R score of 2 and over 11% of children who were not in any pain reported FPS-R scores of 4, 6, 8 or 10. These reports suggest that FLACC and FPS-R evaluation cannot distinguish between pain and the emotional aspects of distress, as noted by Blount and Loiselle (2009).

We wish to highlight limitations and future directions. First, the lack of a control condition prohibits attributions of low distress to qualities of the device or behavioral program. However, given the sensitivity of the eardrum, it is challenging if not unethical to conduct tympanostomy absent anesthesia and behavioral support for pediatric patients. However, dismantling studies might help identify key intervention ingredients. Second, generalizability of findings is limited given our inclusion and exclusion criteria which restricted the patient population to children with anatomy compatible with safe use of the devices and anesthetic, and with compliant behavior for the office procedure. Future research might evaluate whether behavioral strategies might be effective for more challenging patients. Third, two of the authors were funded for work on the project, which could have introduced investigator bias. That said, all data coding was conducted by researchers who were blind to study hypotheses. Given that the behavioral program appeared to be helpful to some but not all children, we encourage researchers in this area to assess additional unique characteristics (e.g., coping styles, temperament) to advance the field in matching intervention components and strategies to individuals.

Myringotomy with tympanostomy tube placement is the most common pediatric surgery, and it is a fairly simple and brief procedure. Unfortunately, the current norm is to use general anesthesia, which invites a host of potential behavioral and other problems for young patients (e.g., Kain et al., 1999; Zhang et al., 2015). The current findings in conjunction with Lustig et al. (2020) indicate that the Tula System and behavioral program allow pediatric patients to receive in-office tympanostomy tube placement without general anesthesia, without sedation, and without mechanical restraints, and with minimal distress.
